# Development of a tissue-engineered skin model with epidermal, dermal and hypodermal components

**DOI:** 10.1007/s44164-023-00058-9

**Published:** 2023-09-21

**Authors:** V. L. Workman, A-V. Giblin, N. H. Green, S. MacNeil, V. Hearnden

**Affiliations:** 1 Department of Materials Science and Engineering, University of Sheffield, Sheffield, UK; 2 Department of Plastic Surgery, Sheffield Teaching Hospitals NHS Foundation Trust, Sheffield, UK

**Keywords:** Tissue engineering, Skin, Adipose, Hypodermis, Cytokeratin, Rete ridges, Dermal-epidermal junction

## Abstract

Tissue-engineered models of skin have evolved over the past 50 years, have successfully been translated to clinical use and continue to be improved using new technologies. However, very few of these constructs incorporate a hypodermal component. The hypodermis is critical to skin homeostasis, skin function and many skin diseases, but our understanding of the hypodermis is limited in comparison to our knowledge of the epidermis and dermis, in part due to a lack of suitable in vitro models.

The purpose of this study was to develop and characterise a tissue-engineered model of skin consisting of epidermal, dermal and hypodermal layers, namely a trilayer skin model. Models were produced by culturing human keratinocytes and fibroblasts on decellularised human dermis in combination with explanted human adipose tissue.

Bilayer models of skin, comprising of an epidermis and dermis, had a thicker epidermal component compared to trilayer models but exhibited similar cytokeratin expression patterns (AE1/AE3 and cytokeratin 14). Addition of adipose tissue improved the appearance of the dermal-epidermal junction, increased the number of rete ridge-like features and cells maintained similar levels of proliferation (Ki-67) compared to native tissues over 28 days in culture.

This technique enabled us to create a physiologically relevant model of human skin with representative morphology across the hypodermis, dermis and epidermis. This model maintained native extracellular matrix architecture, contained a heterogeneous population of cells and has the potential to be applied to a range of different applications where research questions require the inclusion of a hypodermis.

**Supplementary Information:**

The online version contains supplementary material available at 10.1007/s44164-023-00058-9.

## Introduction

Engineering skin substitutes has been possible since the 1980s with numerous different approaches available to culture physiologically relevant and morphologically representative constructs of human skin [1]. The construction and design of tissue-engineered skin models range in complexity from simple cultured epithelial sheets, comprised of only keratinocytes, to complex constructs incorporating a variety of cells, scaffolds and exogenous factors.

The clinical translation of tissue-engineered skin for the treatment of burns and other skin damage has been successful and has demonstrated patient benefit over alternative therapies [1]; however, the logistical, financial and regulatory challenges involved mean that despite scientific advances, tissue-engineered skin is still rarely used in clinical practice [2]. Where tissue-engineered skin constructs have arguably had a greater impact is in their use as in vitro models to answer specific research questions. Engineered skin models have been used effectively to study skin physiology, drug metabolism [3], disease [4], ageing [5] and drug delivery as well as epithelial morphogenesis [6], skin contraction [7] and epithelial/mesenchymal crosstalk [8]. These skin models are now broadly accessible to researchers which have enabled research questions to be answered with human-specific tissues and cells reducing our reliance on unsuitable animal models. However, there are limitations in current models.

One aspect of skin anatomy which has received little attention is the hypodermal component. It is now apparent that the hypodermis and the heterogeneous cells found within it are integral to skin physiology, wound healing and disease pathology through paracrine and endocrine signalling and cell crosstalk [9]. Our understanding of the communication between cell populations in the hypodermis with keratinocytes and fibroblasts is lacking in comparison to our advanced understanding of keratinocyte and fibroblast crosstalk because the majority of tissue-engineered skin constructs do not include a hypodermal component or adipose-derived cells [10]. Incorporating cell populations from the hypodermis has the potential to improve the relevance of engineered tissues and to allow a greater breadth of skin function to be investigated.

New tissue-engineered models which incorporate some features of the hypodermis have been developed in recent years. Sugihara et al. developed trilayer skin constructs comprising of rat keratinocytes, fibroblasts and mature adipocytes cultured on collagen gels [10], and Huber et al. demonstrated that mature adipocytes could improve the morphology of the epidermis [11]. Culturing mature adipocytes is challenging as a result of their buoyancy in culture medium, fragility, specific media requirements and dedifferentiation in culture [12]. An alternative approach is to develop a hypodermis equivalent using multipotent adipose-derived stromal cells (ADSCs). These models can be cultured scaffold free using self-assembly [13] or with scaffolds [14]. While both techniques have created models which incorporate mature adipocytes, it should be remembered that adipose tissue is a highly heterogeneous tissue with diverse cell populations which all contribute to hypodermal function [15].

As well as a lack of the hypodermis, many existing models also lack features of the dermal-epidermal junction such as rete ridges and a basement membrane. Evidence is emerging about the role of adipocytes, preadipocytes and ADSCs on the dermal-epidermal junction and the influence of these cells on rete ridge formation [16] and epidermal stratification and differentiation [10].

In this study, we aimed to develop a reproducible method to generate trilayer human skin models which incorporated key features of skin biology including a mature epidermis, adipose tissue component and a clear dermal-epidermal junction. We aimed to create models that can be applied to answer specific research questions which involve the hypodermis and where the hypodermis plays a critical role, for example, diabetes, obesity, cancer and wound healing. Using a combination of in vitro and ex vivo culture techniques with human tissues and cells, we aimed to create tissue-engineered skin constructs which were biochemically, mechanically and physiologically relevant to native human skin while also being accessible and reproducible by other researchers.

## Materials and methods

### Ethics

Waste adipose tissue and human skin was collected from routine surgical procedures, with written informed consent, following a protocol approved by the NHS research ethics committee (ref: 15/YH/0177 & 21/NE/0115). All tissue was obtained and used on an anonymised basis from Sheffield Teaching Hospitals NHS Trust, Directorate of Plastic, Reconstructive Hand and Burns Surgery. Samples were stored at room temperature for up to 24 h post-excision before processing.

### Skin cell culture

Fibroblasts and keratinocytes were isolated from skin according to the methods described by Ghosh et al. [17]. Keratinocytes were used between passages 1 and 4 and fibroblasts between passages 4 and 11.

Keratinocytes were cultured in Green’s medium—Dulbecco’s Modified Eagle Medium (DMEM) (Merck, Dorset, UK) and Ham’s F12 (Merck, Dorset, UK) medium in a 3:1 ratio supplemented with 10% foetal calf serum (FCS) (PAN Biotech, Dorset, UK), 10 ng/ml epidermal growth factor (EGF) (Bio-Techne, Oxfordshire, UK), 0.4 mg/ml hydrocortisone (Merck, Dorset, UK), 1 × 10 ^−10^ mol/l cholera toxin (Merck, Dorset, UK), 1.8 ×10 ^−4^ mol/l adenine (Merck, Dorset, UK), 5 mg/ ml insulin (Scientific Laboratory Supplies, Nottingham, UK), 5 mg/ml transferrin (Merck, Dorset, UK), 2 × 10 ^−3^ mol/l glutamine (Merck, Dorset, UK), 2 × 10 ^−7^ mol/l triiodothyronine (Merck, Dorset, UK), 0.625 μg/ ml amphotericin B (Merck, Dorset, UK), 100 IU/ml penicillin and 100 mg/ml streptomycin (Merck, Dorset, UK). The fibroblasts were cultured in DMEM supplemented with 10% FCS, 2 ×10 ^−3^ mol/l glutamine, 0.625 mg/ml amphotericin B and 100 IU/ml penicillin and 100 mg/ml streptomycin.

### Preparation of acellular de-epidermised dermis

Donor skin for de-epidermised dermis (DED) preparation was obtained from ETB-BISLIFE, The Netherlands. The glycerol-preserved skin was prepared as described in Deshpande et al. [18]. DED from a single donor was used for each biological repeat, wherever possible cut from the same skin sheet. The DED was cut into squares approximately 15 × 15 mm, and the papillary surface was orientated uppermost in 6-well plates (Corning, Flintshire, UK).

### Production of tissue-engineered skin composites (bilayer models)

Each experiment contained triplicate replicates and was repeated three times (using cells from different skin donors). Keratinocytes (3 × 10 ^5^ cells) and fibroblasts (1 × 10 ^5^ cells) were seeded onto the DED using stainless steel rings with an inner diameter of 1 cm and cultured submerged for 24 h to allow cell attachment. After 24 h, the rings were removed and constructs cultured for an additional 24 h submerged before the cell seeded area was cut out and placed on a stainless steel grid to generate an air-liquid interface (ALI). Models at ALI were cultured in Green’s medium for up to 28 days with media changes every 2–3 days.

### Production of tissue-engineered skin composites (trilayer models)

**Fig. 1:**
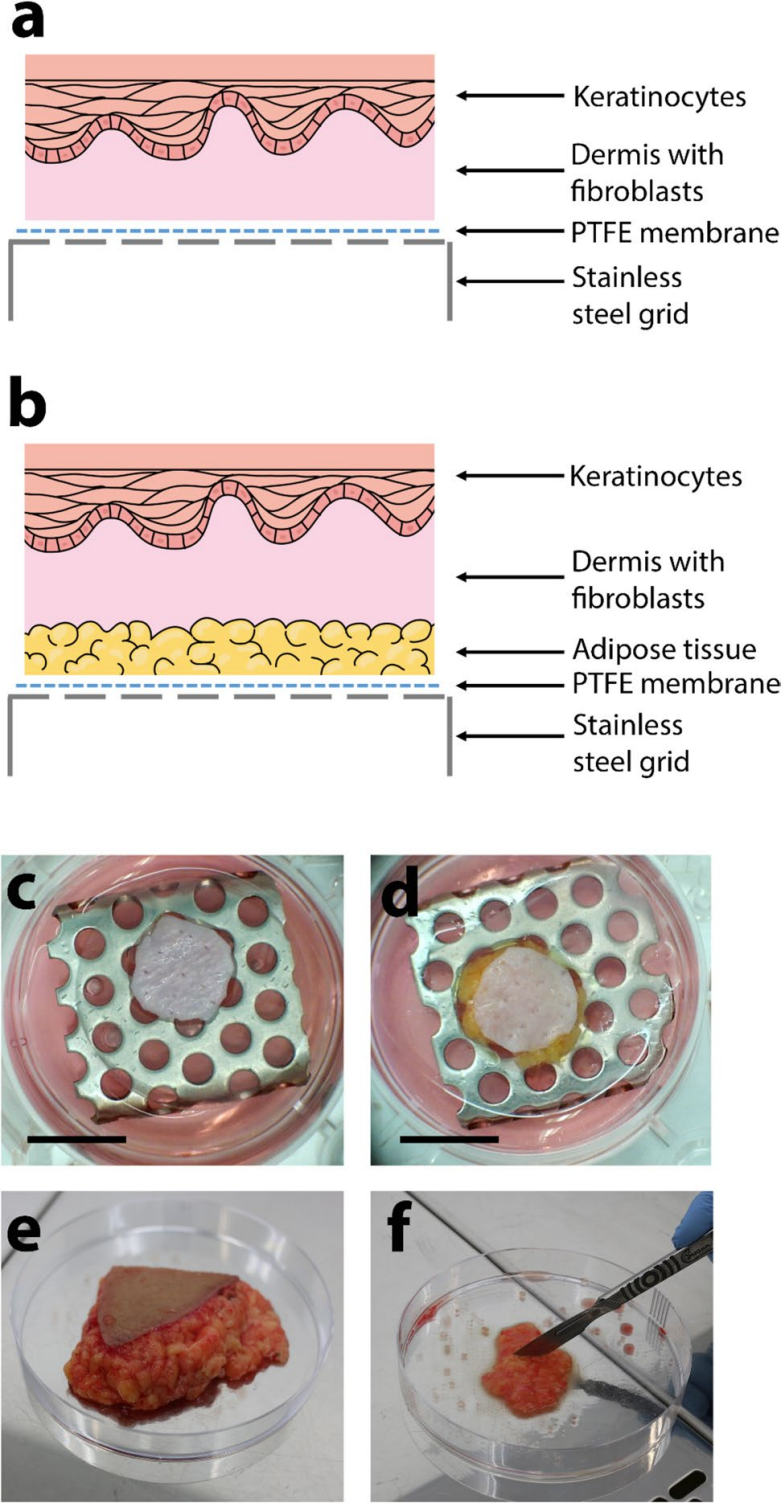
Schematic to demonstrate the components used to construct the bilayer (**a**) and trilayer (**b**) models. A stainless steel grid was used to produce the air-liquid interface and PTFE membrane to stop adipose tissues falling through the holes in the grid. Photographs of bilayer (**c**) and trilayer (**d**) models viewed from above in culture. The dermal papillary surface can be seen in both models, and the transparent PTFE membrane can be observed on top of the grid. In the trilayer models, where adipose tissue was placed beneath the dermis, a small amount of the yellow adipose tissue is visible around the edges of the dermis (**d**). Subcutaneous adipose tissue collected from routine surgery was collected whole (**e**) and minced (**f**) prior to placing underneath the dermis to produce trilayer models. Scale bar = 1 cm

Trilayer models were cultured by first preparing bilayer models as described above. Adipose tissue from patients was minced (Fig. 1f) using a sterile scalpel, and large blood vessels and connective tissue were manually removed. 100 μL of minced adipose tissue was added to each model when it was raised to ALI, and a PTFE membrane (0.45 μm, 25 mm diameter; JHWP02500; Merck-Millipore, Dorset, UK) was placed between the stainless steel grid and the adipose tissue to avoid tissue falling through the holes in the grid (Fig. 1a–d).

### Immunohistochemistry

At the end of the culture period, bilayer and trilayer models were fixed with 10% phosphate-buffered formaldehyde and processed for conventional histology examination with haematoxylin and eosin (H&E) staining. Formalin-fixed paraffin-embedded native skin samples (*n* = 3) were purchased and stained as controls (Insight Biotechnology Ltd, Middlesex, UK). Immunohistochemistry (outlined in Table 1) was carried out using a Dako Omnis autostainer.

**Table 1:** Details of monoclonal antibodies and staining protocol used for DAB immunohistochemistry

	Murine anti-human Ki-67	Murine anti-human cytokeratin	Murine anti-human cytokeratin 14
Clone	MIB-1	AE1/AE3	LL002
Supplier	Dako	Dako	Leica
Antibody type	Ready-To-Use (RTU) Omnis	RTU Omnis	Concentrate
Dewax	Two phase dewax wash IHC	Two phase dewax wash IHC	Two phase dewax wash IHC
Target retrieval IHC	Low pH (Dako Omnis)	High pH (Dako Omnis)	High pH (Dako Omnis)
Primary antibody	20 m	10 m	20 m
Endogenous enzyme block	EnVision FLEX peroxidase-blocking reagent (Dako Omnis) for 3 m		
Labelled polymer	EnVision FLEX/HRP (Dako Omnis) for 20 m		
Substrate chromogen	EnVision FLEX Substrate Working Solution (Dako Omnis) for 5 m		
Counterstain	Haematoxylin (Dako Omnis) for 3 m		

### Image capture and image analysis

Brightfield images of stained slides were taken using an upright compound microscope (Olympus CX43) and Euromex HD-Ultra camera (both supplied by Best Scientific, Wiltshire, UK). ImageJ (Version 1.53c) was used to measure epidermal thickness (comprised of cornified and nucleated layers) and width and depth of rete ridge-like features (Supplementary Figure 1).

### Statistical analysis

Replicate data within each experimental condition was presented as mean values ± standard deviation. A 2-way ANOVA, with Tukey’s correction for multiple comparisons, was used to evaluate statistical significance. Significant values are indicated as *p < 0.01, **p < 0.001 and ***p < 0.0001.

## Results

Bilayer and trilayer skin models were cultured in parallel to investigate the effects of adding adipose tissue to the structure of the tissue-engineered skin. Results were compared to native skin samples.

**Fig. 2:**
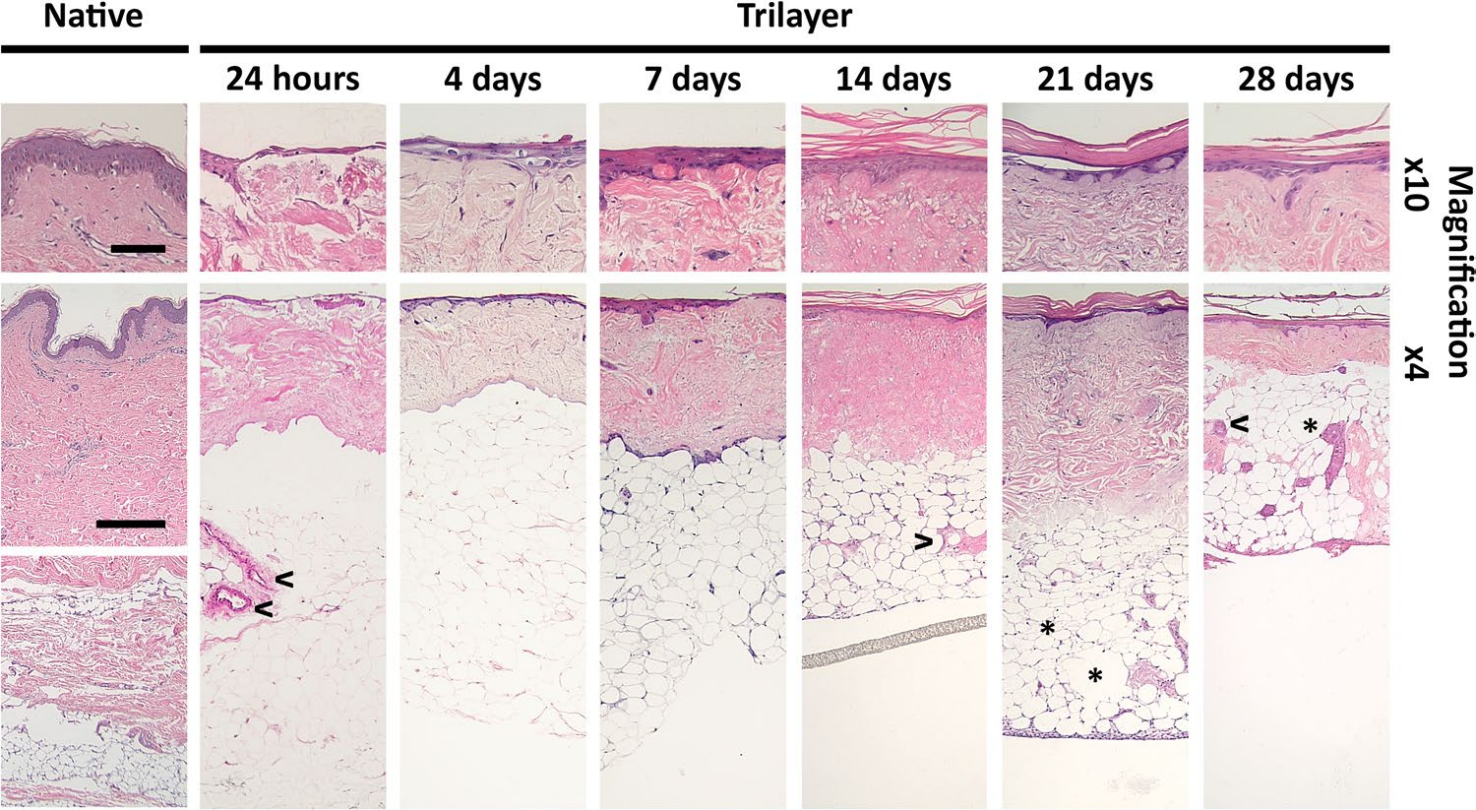
Representative histology images showing development of the trilayer model over 28 days. Tissue sections through native skin or the trilayer model were stained with haematoxylin and eosin. Cell nuclei are stained in purple/blue, and plasma proteins, collagen and keratins are stained in pink. The PTFE membrane used in culture can be seen in the bottom image at 14 days. Top panels (× 10 magnification) scale bar = 100μm. Bottom panels (× 4 magnification) scale bar = 500μm. **>** indicates vascular structures. * indicates changes in adipocyte structure

The morphological features of trilayer models were assessed following culture at ALI for 24 h, 4, 7, 14, 21 and 28 days. All trilayer models had an architecture comparable to native human skin and comprised of the three layers expected: a stratified epidermis, a dermal layer containing fibroblasts and a hypodermal layer (Fig. 2). Keratinocytes formed a single layer on the surface of the dermis after 24 h, and these keratinocytes began to stratify after 4 days of culture at ALI (Fig. 2). From day 4 onwards, the keratinocytes showed evidence of differentiation and keratin production. Between day 7 and day 14, a stratified epidermis was formed with a superficial keratinised layer above a spinous layer.

The adipose tissue added beneath the DED can be seen in the images for all time points. A distinct gap between the adipose tissue and the dermal layer of the model was observed at 24 h and 4 days of culture at ALI. After 7 days of culture at ALI and for the remainder of the experiment, the adipose tissue was contiguous and well attached to the dermal layer (Fig. 2). As the hypodermal component in this model was an ex vivo culture of the whole adipose tissue, other components of the adipose tissue including blood vessels (labelled with < in Fig. 2) and the associated cell populations were visible in some samples and persisted throughout the culture period. The majority of adipocytes had associated nuclei and maintained a consistent size up to 14 days of culture at ALI. In some samples, select adipocytes appear to have fused and may have lost integrity by the later time points (labelled with * in Fig. 2).

**Fig. 3:**
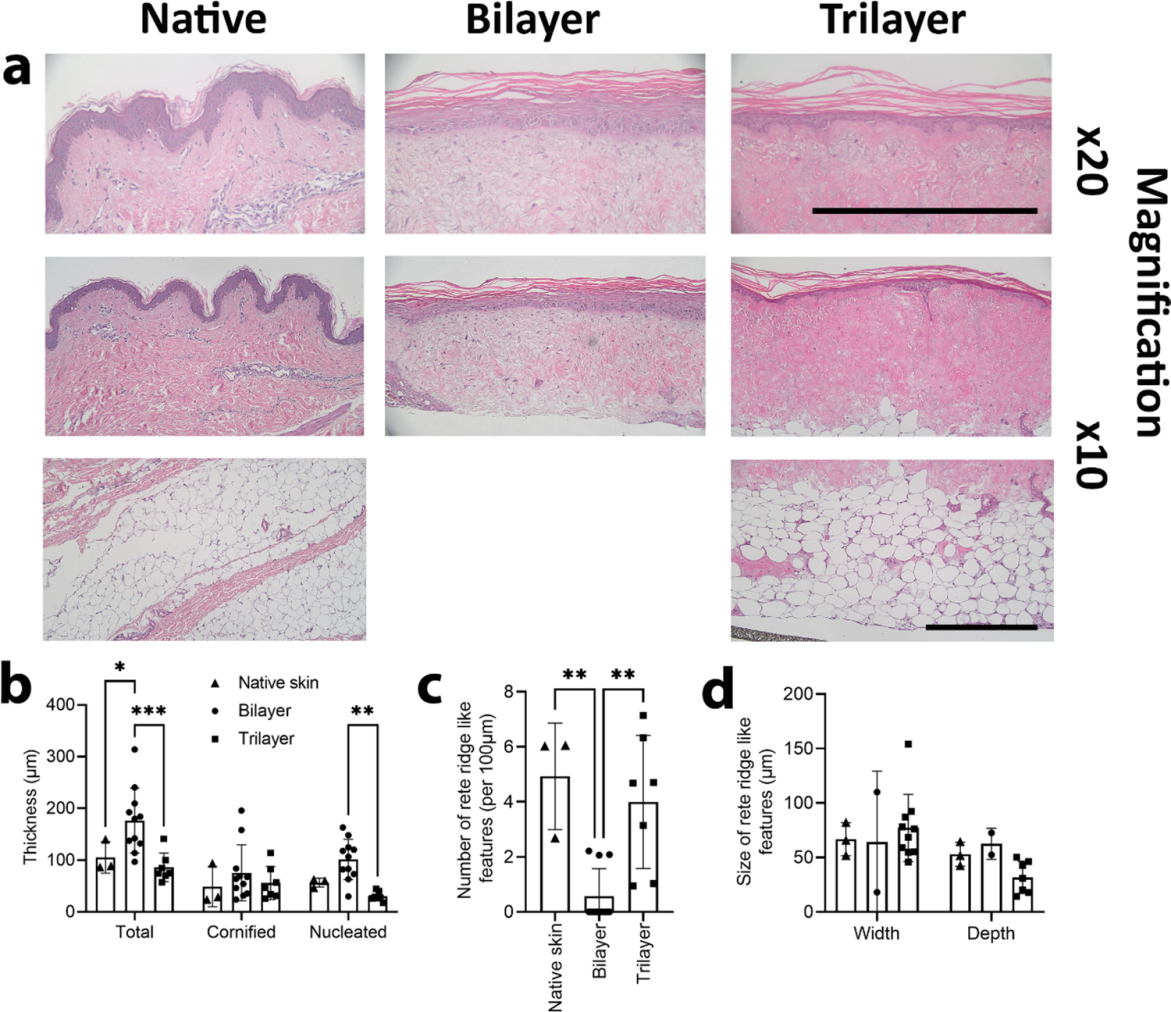
**a** Representative images showing native skin compared with bilayer and trilayer models after 14 days in culture. The epidermis and hypodermis are shown. Scale bar = 500μm. **b** The thickness of the nucleated cell layer in the epidermis of bilayer and trilayer models after 14 days in culture was compared with native skin (*n* = 3). **c** Number and **d** depth and width of rete ridge-like features present in bilayer and trilayer models after 14 days in culture were compared with native skin (*n* = 3). Triangles = native skin, circles = bilayer, squares = trilayer. *p < 0.01, **p < 0.001, ***p < 0.0001

Total epidermal thickness, comprising cornified and nucleated layers, was quantified (Supplementary Figure 1) for bilayer and trilayer models cultured at ALI for 14 days. Native skin (female, abdominal) was used as a comparator (Fig. 3). The average epidermal thickness in trilayer models was approximately 86 μm, which was comparable to the value observed in native skin (105 ± 30 μm). In contrast, the epidermis of the bilayer models was almost twice as thick as native skin (177 ± 63 μm). The increased thickness was predominantly as a result of an increase in the nucleated portion of the epidermis in the bilayer models (Fig. 3b).

There were approximately four rete ridge-like features per 100 μm (linear distance) in native skin and trilayer models (Fig. 3c). In contrast, the bilayer models had less than one rete ridge-like feature per 100 μm (with many samples having no discernible rete ridges). The difference in the number of rete ridge-like features was statistically significant despite the large variability. There was no significant difference in the width and depth of rete ridge-like features, which were approximately 60 μm in all samples; this was comparable to native skin (Fig. 3d).

**Fig. 4:**
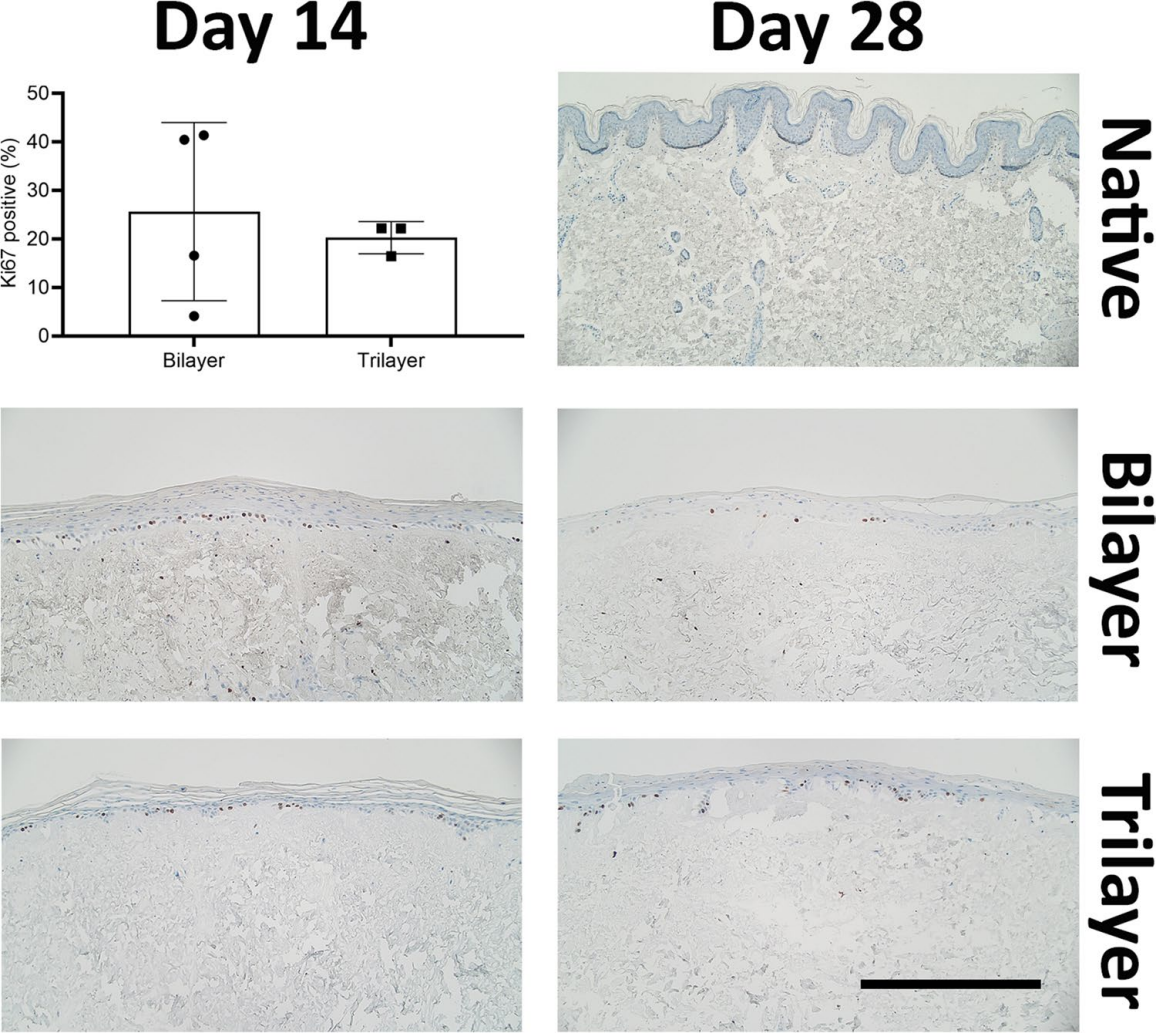
Representative immunohistochemistry images showing the presence of proliferating cells stained with Ki-67. Tissue sections through native tissue, bilayer model and trilayer model were taken 14 days and 28 days after culturing at ALI. Scale bar = 500 μm

**Fig. 5:**
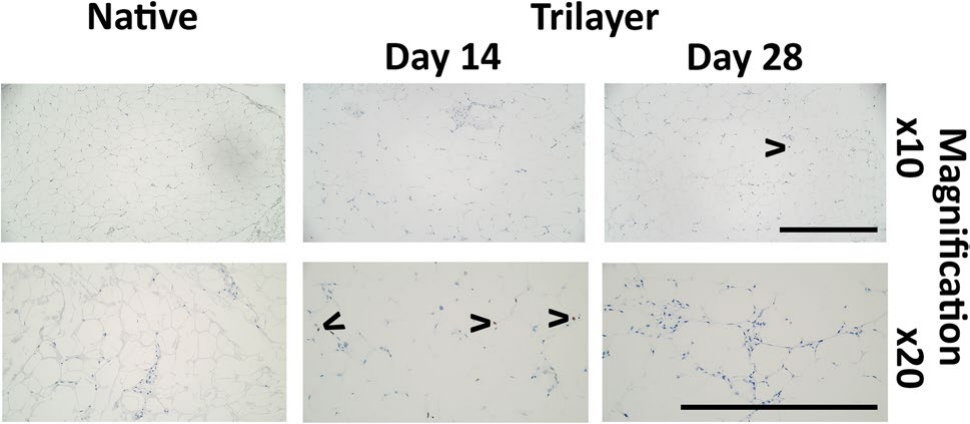
Representative immunohistochemistry images showing the presence of proliferating cells stained with Ki-67 in the hypodermal component. The native hypodermis was compared to the trilayer model after 14 days or 28 days of culture at ALI. < indicates Ki67 positive cells. Scale bar = 500 μm

To assess the effect of the hypodermal component on cell proliferation in basal keratinocytes, sections of bilayer and trilayer models collected at 14 and 28 days of culture were stained with Ki-67 and compared to native skin (Fig. 4). Proliferative cells were present in the basal layer of the epidermis in all samples, at both time points. The proportion of Ki-67 positive cells in the epidermis was approximately the same (25%) in the bilayer and trilayer models; however, the standard deviation was smaller for the trilayer models compared to the bilayer models (Fig. 4). A low proportion of cells within the dermis (Fig. 4) and hypodermis of native skin (Fig. 5) were Ki-67 positive with similar expression patterns seen in the dermis and hypodermis of the bilayer and trilayer models (Figs. 4 and 5). The expression of Ki-67 in the hypodermal component at day 14 appeared to be slightly higher than in both native skin and day 28 samples; however, the expression was too low to draw conclusions.

**Fig. 6:**
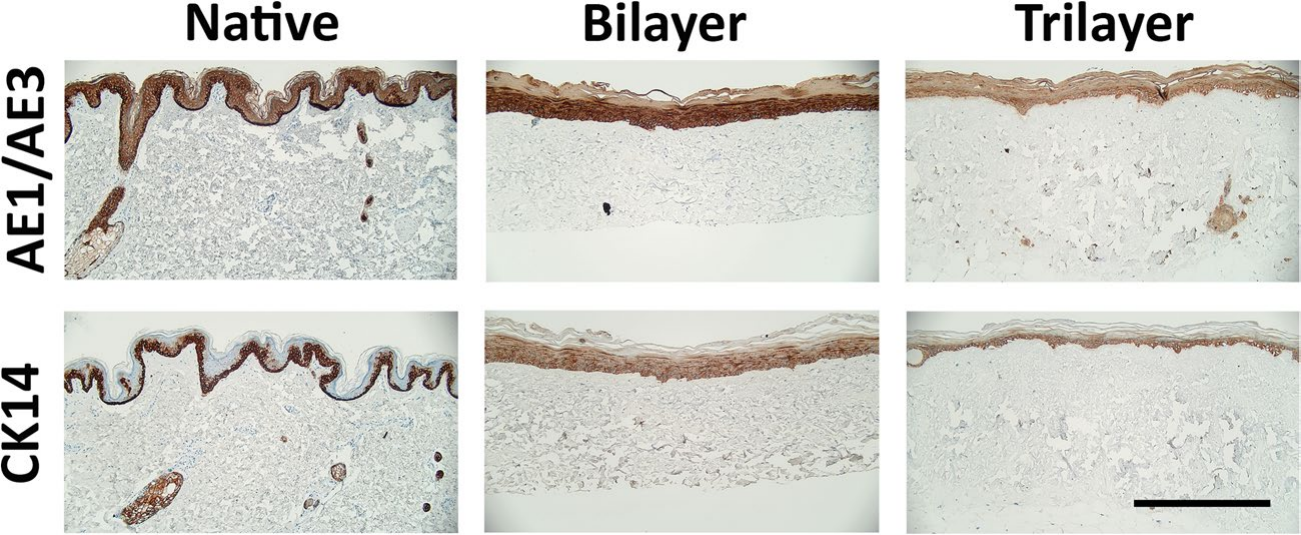
Representative immunohistochemistry images showing the presence of AE1/AE3 (top panel) and CK14 (bottom panel) using DAB staining comparing native tissue, bilayer model and trilayer model. Samples were taken after 14 days at ALI. Scale bar = 500 μm

To determine the differentiation status of keratinocytes forming the epithelium, samples were stained with the pan-cytokeratin marker AE1/AE3 [19] and cytokeratin 14 (CK14), a marker of basal keratinocytes [20]. After 14 days of culture at ALI, samples of both bilayer and trilayer models were seen to have a similar expression compared to native skin for AE1/AE3 and CK14 (Fig. 6). In native skin and the bilayer models, AE1/AE3 staining was stronger in the basal layers than in the stratum corneum. In the trilayer model, there was still stronger staining in the basal layer than the stratum corneum, but the staining in the basal layer was less strong than that observed in native skin or bilayer models. The basal layers of the epidermis in all samples had CK14 expression with some suprabasal expression also observed in the bilayer and trilayer models (Fig. 6).

## Discussion

In this study, we have shown that combining cultured human keratinocytes, cultured human fibroblasts and ex vivo human adipose tissue with a de-epidermised skin scaffold results in a trilayer human skin construct which is morphologically similar to native skin. The addition of minced adipose tissues to our previously developed bilayer tissue-engineered skin models produced models with a viable well-attached hypodermal component which could be cultured for up to 28 days.

The model presented here utilised waste adipose tissue, generated from routine surgeries. The ex vivo culture of this human adipose tissue provided a heterogeneous population of hypodermal cells, in contrast to previous models which have used cultured, mature adipocytes [11] or differentiated ADSCs [13, 14, 21]. The hypodermis in the model presented here adheres well to the dermis, has a high density of mature adipocytes and maintains the adipose tissue morphology found in native skin.

Although adipocytes comprise over 90% of adipose tissue by volume, they make up less than 50% of the cellular content [15]. The remaining cells within adipose tissue include stromal cells (including stem cells and progenitors), endothelial cells, leukocytes, lymphocytes and other immune cells. The endothelium and perivascular region within adipose tissue provides a growth niche for adipocyte progenitors [22], plays a key role in obesity [23] and is required for revascularisation [24]. Vessel-like structures and endothelial cells from the explanted adipose tissue can be observed in the hypodermal layer of the model at all time points investigated (shown with >). This demonstrates that some microenvironmental features are retained within the native adipose ECM in these models. Further investigation is warranted to determine how functional and viable these populations of cells remain during the culture period.

ADSCs cultured and expanded in vitro rapidly change their paracrine secretions and gene expression [25] while the culture of adipocytes requires technically challenging culture conditions [12]. By using an explant culture approach, we have been able to keep ADSCs and adipocytes in their endogenous ECM environment, limiting culture-induced changes in these cells and also saving time for the establishment of models. To investigate the role of the hypodermal layer in skin homeostasis and disease, the inclusion of heterogeneous adipose tissue is expected to be beneficial in comparison to models which include mono cultures of ADSCs or adipocytes.

Proliferative cells (positive for Ki-67) were found in small numbers within the hypodermis after 28 days of culture, which was comparable to native skin. Explanted hypodermal tissues retained structural integrity through the experiment with some small changes in adipocyte size and integrity at the 28-day time point. Previous studies by Schopow et al. showed that adipose tissue could survive in slice culture for 14 days (measured through perilipin A expression) [26]. In this study, we minced adipose tissues to improve nutrient diffusion and to support the set-up of constructs on PTFE membranes. Lipoaspirate has also been used (data not shown) resulting in very similar models, demonstrating the versatility of this model based on available human tissue.

Our trilayer skin model was constructed over 3 days and matured over the following 7 days at ALI. Once the epithelium had stratified, the trilayer skin model could be studied for a further 3 weeks. Proliferating cells were still detectable in all layers of our skin model after 28 days at ALI and an intact epidermis was maintained. This is an improvement on previous trilayer models which have lost epidermal and adipocyte integrity after 7 days in culture [11] or which take many weeks to set up [13].

The culture conditions needed to generate a stratified, keratinised epidermis in vitro are well established, and the role of fibroblast crosstalk and physical stimuli is well accepted [1]. An area which has been less well investigated is how the hypodermis and cells within it affect epidermal morphogenesis. In this study where bilayer and trilayer models were cultured in parallel with cells, adipose tissue and DED from the same donors, it was shown that models cultured without adipose tissue had a thicker epidermis.

The thickness of the human skin varies based on many factors including age, ethnicity and anatomical location [27]. Native epidermal thickness has been reported to range from 77 to 267 μm [27], with our measurements taken from native skin (105 μm) and trilayer model (86 μm) in agreement with this. In contrast, the average epidermal thickness of the bilayer models was almost twice as thick (177 μm) with some models having an epidermis as thick as 300 μm. The majority of the difference in epidermal thickness was seen in the nucleated rather than cornified component of the epidermis. In addition, there were variable levels of Ki-67 expression in the bilayer models suggesting the adipose component may be modulating proliferation of keratinocytes as previously shown [10, 14].

The addition of adipose tissue also influenced the dermal-epidermal junction and epithelial morphology. Structural features such as rete ridges are found throughout the human skin, and these interdigitations increase the surface area of the dermal-epidermal junction and thus enhance adhesion between the layers, nutrient supply and mechanical strength. In addition, rete ridges are thought to provide a niche for epidermal stem cells, although it is unclear where they reside (as reviewed in [28]). Rete ridges are rarely seen in tissue-engineered skin models as their formation requires complex and topographical cues. However, there are examples of scaffold designs which incorporate topographical cues to stimulate rete ridge formation which have been shown to increase the metabolic activity of keratinocytes [29].

In this study, bilayer models made with DED did not form as many rete ridge-like projections compared to the trilayer models cultured with explanted adipose tissue. A previous elegant study by Aoki et al. showed that bone marrow stromal cells but not preadipocytes induced keratinocytes to form rete ridge-like structures suggesting it may be the ADSCs present in our hypodermal component which are responsible for the rete ridge-like projections. Here, we have shown that with a combination of DED and adipose tissue, we were able to generate models with rete ridge-like projections of a similar size and density to native skin.

Cytokeratin 14 is expressed in the stratum basale of the native skin; however, in our models, the expression was observed throughout the nucleated portion of the epidermis in both bilayer and trilayer models. This pattern of expression, in the basal and suprabasal layers of engineered epidermis, has previously been reported and is likely due to the way epidermal cells mature during stratification in vitro [11, 16].

The primary aim of this study was to develop an engineered tissue to support research into a range of skin pathologies and to reduce the use of unsuitable in vivo models. This model is expected to have value in a range of studies where the hypodermis is important (such as research into diabetes, obesity, ageing and cancer) while also providing a tool to develop novel therapeutics and diagnostic technologies in vitro. We are not proposing that this trilayer engineered skin is a suitable graft for clinical use to treat full thickness defects. Vascularisation and nutrient diffusion through the adipose tissue are expected to be a significant challenge, and we would expect graft failure to occur if these thicker tissues were applied to a wound bed. It is hoped that the model presented here is a useful and accessible tool to improve our understanding of skin biology and the role of the hypodermis in skin homeostasis.

This model relies on the use of freshly excised human tissues which can be difficult to access, and we have observed patient-to-patient variability. While this is a limitation of this approach, we believe using primary human tissues and cells presents our best opportunity to generate physiologically relevant tissues and opens up the possibility to generate personalised or disease-specific models using tissues and cells from select individuals with different pathologies.

Decellularised tissues have biological and anatomical variation making them less reproducible than synthetic scaffolds; however, the biological relevance to native tissue is extremely high, making this model suitable for studies into matrix biomechanics and research questions where the extracellular matrix (ECM) is crucial. The dermal scaffold used in this model comprises of de-epidermised dermis (DED) which undergoes a mild decellularisation process (sodium chloride treatment and mechanical scraping), retaining a native ECM composition, orientation and basement membrane [30]. The basement membrane has previously been shown to support keratinocyte attachment, helping them to maintain stemness [31, 32]. In this study, we have also shown that the underneath of the DED is able to adhere to adipose tissues supporting signalling between skin components and ensuring tissue integrity. The morphology and architecture of the tissues produced in this study have an ECM which more closely resembles native human skin tissue compared to models constructed with collagen gels, self-assembly techniques or synthetic scaffolds.

We have added a heterogeneous population of cells through the use of explanted minced adipose tissues; however, in this study, we have not investigated which cell populations remain functional and viable throughout the culture period; this would be an interesting area of future investigation. Adding an immune component to tissue-engineered models has long been held as an aspiration by many in the field [33]; therefore, it would be interesting to see if immune cells found in the adipose tissue such as tissue-resident macrophages or neutrophils persist in this model.

## Conclusions

The model presented here demonstrates that tissue-engineered models of the human skin can be produced, which morphologically resemble full-thickness native skin, by culturing keratinocytes and fibroblasts on a decellularised dermis in combination with minced adipose tissue. This model can be generated reproducibly, and viable cells can be observed after 28 days at air-liquid interface. This model is relatively quick to establish and presents the opportunity to study full-thickness skin including a hypodermal component which has a heterogeneous population of cells, a native ECM and a density and distribution of mature adipocytes similar to native skin.

In this study, we have shown that the addition of adipose tissue altered the topography of the dermal-epidermal junction and affected epidermal morphogenesis. This model has the potential to further increase our understanding of the role of adipose derived-cells and tissues on cutaneous biology while also providing a valuable tool for drug discovery and translational research.

## Supplementary information

Supplementary Figure 1: Demonstration of how measurements were made for nucleated, cornified and total epidermal thickness and for rete ridge-like features

High resolution image (TIF 831 kb)
