# Supplementary figures and images for: Development of a tissue-engineered skin model with epidermal, dermal and hypodermal components
Source: In Vitro Model. 2023 Sep 21;2(6):297–306. doi: 10.1007/s44164-023-00058-9 (PMC11756454; doi:10.1007/s44164-023-00058-9)

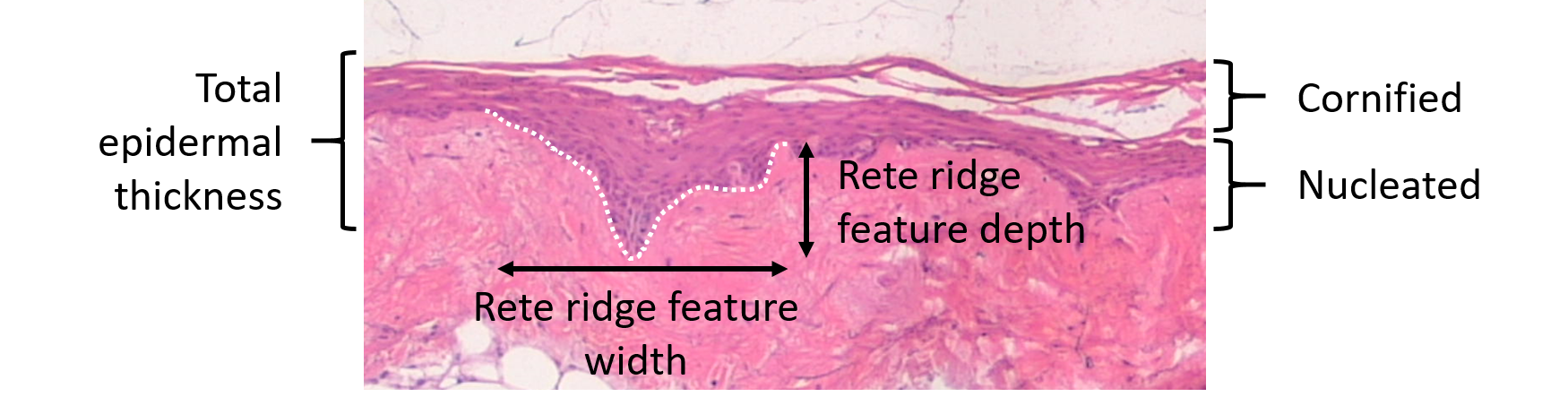

Supplement: Supplementary file 1 — Demonstration of how measurements were made for nucleated, cornified and total epidermal thickness and for rete ridge-like features [file 44164_2023_58_Fig1_ESM.png]

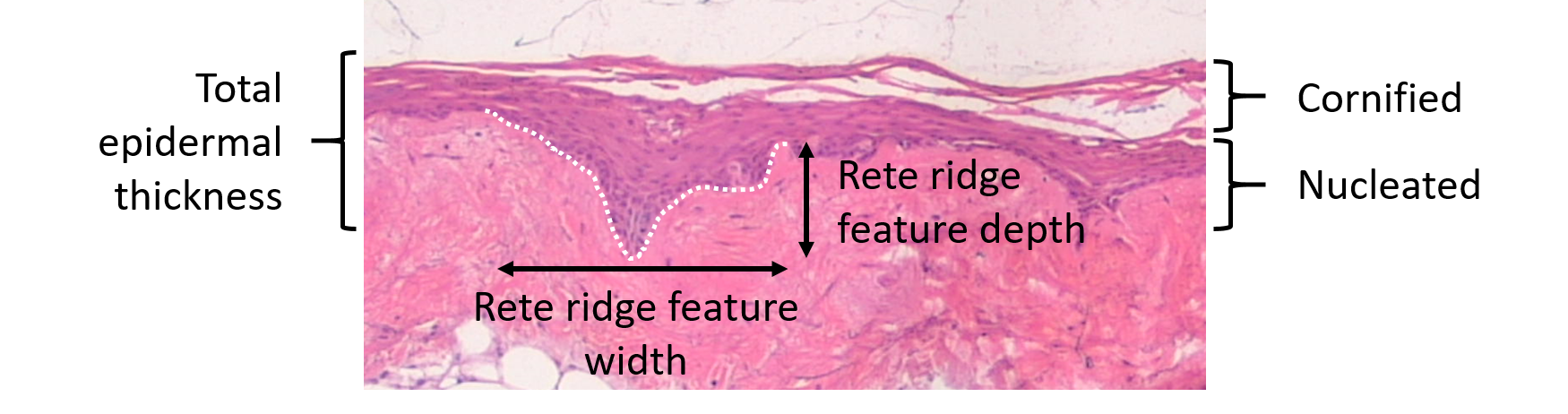

Supplement: Supplementary file 2 — High resolution image (TIF 831 kb) [file 44164_2023_58_MOESM1_ESM.tif]
